# “Gut Microbiota-Circadian Clock Axis” in Deciphering the Mechanism Linking Early-Life Nutritional Environment and Abnormal Glucose Metabolism

**DOI:** 10.1155/2019/5893028

**Published:** 2019-08-27

**Authors:** Liyuan Zhou, Lin Kang, Xinhua Xiao, Lijing Jia, Qian Zhang, Mingqun Deng

**Affiliations:** ^1^Key Laboratory of Endocrinology, Translational Medicine Center, Ministry of Health, Department of Endocrinology, Peking Union Medical College Hospital, Peking Union Medical College, Chinese Academy of Medical Sciences, Beijing, China; ^2^Department of Endocrinology, The Second Clinical Medical College of Jinan University, Shenzhen People's Hospital, Shenzhen 518020, China

## Abstract

The prevalence of diabetes mellitus (DM) has been increasing dramatically worldwide, but the pathogenesis is still unknown. A growing amount of evidence suggests that an abnormal developmental environment in early life increases the risk of developing metabolic diseases in adult life, which is referred to as the “metabolic memory” and the Developmental Origins of Health and Disease (DOHaD) hypothesis. The mechanism of “metabolic memory” has become a hot topic in the field of DM worldwide and could be a key to understanding the pathogenesis of DM. In recent years, several large cohort studies have shown that shift workers have a higher risk of developing type 2 diabetes mellitus (T2DM) and worse control of blood glucose levels. Furthermore, a maternal high-fat diet could lead to metabolic disorders and abnormal expression of clock genes and clock-controlled genes in offspring. Thus, disorders of circadian rhythm might play a pivotal role in glucose metabolic disturbances, especially in terms of early adverse nutritional environments and the development of metabolic diseases in later life. In addition, as a peripheral clock, the gut microbiota has its own circadian rhythm that fluctuates with periodic feeding and has been widely recognized for its significant role in metabolism. In light of the important roles of the gut microbiota and circadian clock in metabolic health and their interconnected regulatory relationship, we propose that the “gut microbiota-circadian clock axis” might be a novel and crucial mechanism to decipher “metabolic memory.” The “gut microbiota-circadian clock axis” is expected to facilitate the future development of a novel target for the prevention and intervention of diabetes during the early stage of life.

## 1. Introduction

According to the latest data from the International Diabetes Federation (IDF), there were approximately 425 million adults with diabetes mellitus (DM) worldwide in 2017. By 2045, this number is expected to increase to 629 million, which will bring a tremendous economic burden to the world. In China, the number of diabetic patients has reached 114.4 million, making it the country with the highest prevalence of diabetes in the world. Type 2 diabetes mellitus (T2DM) accounts for more than 90% of the diabetes in the diabetic population [1]. T2DM is a chronic complex disease characterized by high levels of blood glucose, insulin resistance, and relative insulin deficiency. Genes and traditional environmental factors, including obesity and physical inactivity, have been widely recognized in the pathogenesis of T2DM [2], but these factors could not completely explain the current high prevalence and rapid growth of diabetes [3, 4]. Therefore, the etiology and pathogenesis of T2DM are still not fully understood. In recent years, the relationship between the adverse developmental environment in early life and glucose metabolism has gained wide attention from the academic community. “Metabolic memory” and the Developmental Origins of Health and Disease (DOHaD) hypothesis were subsequently proposed [5]. Our previous research [6, 7] and accumulating evidence [8–10] have indicated that an adverse nutritional environment in the uterus significantly increases the risk of chronic metabolic diseases in adulthood. The biological basis of the relationship between the early-life nutritional environment and adult chronic diseases may be the key to the pathogenesis of T2DM. Thus, this review discusses a novel mechanism to elucidate the DOHaD hypothesis.

## 2. Circadian Misalignment Plays an Important Role in Nutrition Intake Disorders and Abnormal Glucose Metabolism

The Nobel Prize in Physiology and Medicine in 2017 was awarded to Jeffrey C. Hall, Michael Rosbash, and Michael W. Young for discovering the molecular mechanisms that control circadian rhythm [11]. The circadian clock, or circadian rhythm, is an intrinsic rhythm formed by the organism's rotation with the earth to adapt to the periodic alterations in the environment. When the environment changes, the body can readjust its own circadian clock by sensing external clues (mainly light). The circadian clock system includes a central circadian clock and peripheral circadian clocks. The central circadian clock is located in the suprachiasmatic nucleus (SCN) of the hypothalamus, which is thought to be the primary pacemaker of circadian rhythm by sensing the light in the environment and integrating the information to form a 24-hour circadian rhythm. In addition, the SCN is also responsible for transmitting signals to the peripheral circadian clock through hormones or synapses and controlling the circadian rhythm of the body. The peripheral circadian clock is widely distributed in tissues, including the intestine, pancreas, heart, liver, skeletal muscles, and kidneys. The peripheral circadian clock is partly controlled by the central circadian clock in circadian rhythm, and at the same time, it has its own oscillator to regulate the function of various tissues and organs [12–14]. Most of the circadian clock components are transcription factors that regulate gene expression. The most widely studied clock genes include *Bmal1* (aryl hydrocarbon receptor nuclear translocator-like, also known as Arntl), *Clock* (circadian locomotor output cycles kaput), *Per1/2/3* (period circadian clock), and *Cry1/2* (cryptochrome). The expression of the clock genes also has a circadian rhythm, which is mainly regulated by a transcription-translation feedback loop. The CLOCK/BMAL1 heterodimer can bind to the enhancer in the promoter region of *Per*, *Cry*, and other genes to initiate the expression of downstream genes. In contrast, the PER/CRY heterodimer can, in turn, inhibit the expression of *Clock* and *Bmal1* to form a regulatory feedback loop for the expression levels of *Clock*/*Bmal1*-*Per*/*Cry* [15]. Furthermore, these clock genes perform circadian rhythm output by regulating the expression of multiple downstream clock-controlled genes. In addition to light, dietary nutrition [16, 17], temperature [18, 19], sleep [19–22], stress [23, 24], and exercise [25–27] all have a regulatory effect on the circadian clock.

It is well known that there is a close relationship between circadian rhythm and metabolism. As early as the 1970s and 1980s, Professor Panda discovered that some patients who had poor glucose response in the evening had no symptoms of DM when they received the same challenge in the morning. Even in healthy people, the rate of glucose metabolism at nighttime meals is also much slower than that at breakfast, indicating that glucose metabolism is associated with circadian rhythm [28]. A large number of clinical studies [29–31] on shift workers and animal experiments [32, 33] have confirmed that circadian rhythm disorders play an important role in the pathogenesis of DM. For shift workers, their sleeping and eating times are disrupted. Then, circadian misalignment occurs, which makes midnight eating possible. However, feeding behavior plays an important role in the nutritional status of the body, which includes nutrition components, nutrition intake, and feeding time. The feeding time is mainly determined by the endogenous time mechanism of the body. In addition, it is also affected by food supply, sense of hunger and satiety, social habits, and convenience. Accumulating evidence in recent years has suggested that the timing of nutrition intake may affect a variety of physiological processes, including sleep-wake cycles, core body temperature, behavior, alertness, and energy metabolism [34, 35]. Animal studies have found that mice fed a high-fat diet (HFD) during the day (sleeping time) gained more weight and had worse glucose tolerance than those fed a high-fat diet during the night (active time). At the same time, the expression of clock genes in adipose tissue and the liver also changed, and the circadian rhythm disorder occurred, while the central circadian clock was not significantly influenced [36–38]. In addition, a moderate mealtime disorder can also lead to disorders of glucose metabolism. A randomized clinical study showed that skipping breakfast significantly increased postprandial blood glucose and decreased insulin and GLP-1 levels compared with consuming three meals a day [39]. The expression of the clock genes in the peripheral blood leukocytes of the breakfast skippers was significantly changed, and the circadian rhythm was disrupted [40]. Animal studies have also demonstrated that skipping breakfast leads to expression disorders of peripheral clock genes and downstream metabolic genes in the liver, while skipping dinner affects lipid metabolism and adipose tissue aggregation [41]. Furthermore, there is a close relationship between the changes in intake of nutrition components and the disorders of circadian clock. Recently, several studies have explored the role of circadian clock in the abnormal glucose metabolism caused by HFD (overnutrition). In the HFD group, the feeding rhythm changed compared with the feeding rhythm associated with the normal diet, with more food eaten during the day (resting period). The expression of clock genes as well as downstream clock-control genes in peripheral circadian clocks such as liver, kidney, adipose tissue, and pancreas changed significantly, leading to disorders of glucose and lipid metabolism [37, 42–45]. In another way, growing evidence showed that modulating the daily period of feeding and fasting, which readjusts the circadian rhythm, could counter the deleterious effects of nutrient imbalance on metabolism [46–48]. Time-restricted feeding (TRF), where food access is restricted to certain hours of the day, is found to have protective effects against HFD or high-fructose diets induced metabolic disorders [49]. Mitchell et al. found that extended daily fasting, independent of the nutrition challenges, could produce metabolic health and longevity benefits in male mice [50]. Thus, regulating the circadian rhythm of food intake could protect against the metabolic disorders induced by adverse nutrient intake. In future, more associated studies need to be done in human to validate these findings.

Therefore, circadian misalignment might be a crucial factor in mediating abnormal nutritional intake and glucose intolerance. In light of the significantly increased risk of metabolic diseases in later life after exposure to an adverse nutritional environment in early life, we further discuss the role of the circadian clock in “metabolic memory.”

## 3. Disorders of Circadian Rhythm Might Be an Important Mechanism Relating Early-Life Nutritional Environment and Abnormal Glucose Metabolism

Early life, including intrauterine development and the neonatal period, is a critical period for fetal growth and development. The early developmental environment has a lasting memory effect that lasts the whole life, called “metabolic memory,” which has been widely accepted and recognized by the academic community. Since Barker first discovered that people with lower birth weight had higher death rates from ischemic heart disease [51], large number of clinical studies [52–57] and animal experiments [7, 58–63] have demonstrated that adverse early-life exposures, such as nutrient restriction or overnutrition, gestational diabetes and maternal obesity and HFD, significantly increased the risk of developing metabolic diseases in later life. However, the precise underlying mechanism by which deciphers the “metabolic memory” is still not fully understood. Some of the recent studies found that maternal obesity and maternal HFD consumption could inhibit and reprogram the expression of clock genes, including *Clock*, *Bmal1*, *REV-ERBα*, *Cry*, and *Per*, in the liver and heart of the offspring, which further led to abnormal glucose and lipid metabolism in offspring and produced long-term memory effects [64, 65]. Mouralidarane et al. demonstrated that the interaction between maternal obesity and a past-natal obesogenic environment increased offspring risk of nonalcoholic fatty liver disease through programming disruptions of 24-h rhythm in clock genes, including *Clock*, *Bmal1*, *Cry2*, and *Per2* in mice [66]. In addition to clock genes, our previous study [58] and other research [67] showed that adverse early-life nutritional challenges programed metabolic diseases in later life; in the meantime, clock-control genes were significantly disrupted, such as *PPARα*, inositol-requiring 1 alpha (IRE1*α*), protein kinase RNA (PKR)-like ER kinase (PERK),and activating transcription factor 6 (ATF6) in the endoplasmic reticulum stress-associated unfolded protein response (UPR) signaling pathways, of which the expressions are rhythmic and play a key role in the link between circadian rhythm and metabolism.

Thus, disorders in circadian rhythm might be a crucial mechanism in linking an adverse nutritional environment in early life and increased risks of metabolic disorders in later life. However, the specific mechanism of the circadian rhythm reprogramming is not yet clear. In light of the significant relationship between gut microbiota and nutrient intake, whether gut microbiota is a crucial factor during this process is unknown. We further discuss the crosstalk between the circadian clock and the gut microbiota in mediating “metabolic memory.”

## 4. The “Gut Microbiota-Circadian Clock Axis” May Be the Key to How an Adverse Nutritional Environment Results in Glucose Metabolism Disturbances

The intestine is the largest immune organ of the human body. As one of the peripheral circadian clock organs, it receives the synchronized information of the central circadian clock. The intestine also has its own oscillator, which is mainly regulated by the nutrition in food [17, 68]. The gut microbiota, with a total weight of 1-2 kg in the intestine, includes more than 1000 species and more than 10^14^ microorganisms. These microorganisms usually have a balanced symbiotic relationship with the host and play an important role in human health. The intestinal microbiota has a variety of important physiological functions. In terms of metabolism, the intestinal microbiota can synthesize the amino acids required by the host, absorb fat and fat-soluble vitamins from the diet, participate in bile acid-related metabolism, help the host digest complex carbohydrates and plants, and produce short-chain fatty acids (SCFAs), such as butyric acid, acetic acid, and propionic acid. In addition, gut microbiota plays important roles in the maintenance of the intestinal epithelial barrier, the regulation of intestinal permeability, and the maturation and regulation of host innate immunity and adaptive immunity, through which it is linked to various organ systems throughout the body [69–71]. More importantly, emerging evidence has showed that the intestinal microbial community interacted with the circadian clock, and disrupting this interaction could result in metabolic diseases [72].

It has been previously found that there are diurnal oscillations in composition and function of gut microbiota itself whose regulation is controlled by host feeding rhythms and the types of food consumed [73, 74]. If the rhythmic feeding times are disrupted, such as host genetic molecular clock deficiency and time-shift-induced jet lag, then aberrant gut microbiota diurnal rhythmicity and dysbiosis occurred. Further transplanting the time-shifted microbiota into germ-free mice led to a significant increase in body adiposity, which implicated that jet lag induced metabolic disorders were transmissible by gut microbiota [72]. Another study also showed that continuous circadian misalignment produced decreased alpha diversity, a higher ratio of Firmicutes/Bacteriodetes, increased intestinal permeability, and changed clock gene expression in intestine of mice fed a high-fat, high-sugar diet [75]. As food intake has been proved to affect the gut microbial community structure and nutrition consumed could regulate peripheral clock rhythm, emerging recent studies have shown that gut microbiota might be responsible for the reprogramming of circadian rhythmicity. Firstly, circadian clock genes, including kaiA, kaiB, and kaiC, have been identified in the *cyanobacterium Synechococcus elongatus PCC 7942* [76]. Secondly, evidence has shown that some bacteria could rhythmically regulate the behavior of host and synchronize with the host [77, 78]. Furthermore, the absence of gut microbiota has been found to disrupt the circadian clock genes, including *Bmal1*, *Cry1*, *Per1*, and *Per2*, of intestinal epithelial cells and liver in germ-free and antibiotic-induced mice [79, 80]. Murakami et al. using fecal transplant model demonstrated that gut microbiota from the HFD fed mice dramatically reprogrammed the liver circadian clock by *PPARγ* [81]. Another study found that gut microbiota regulated body composition through circadian transcription factor NFIL3, which is a significant link among microbiota, host metabolism, and circadian clock [82]. Therefore, the circadian clock influences the composition of gut microbiota, and inversely, the gut microbiota can also regulate the circadian rhythm, which indicates bidirectional communication between gut microbiota and circadian clock (Table 1). In other words, there is a “gut microbiota-circadian clock axis.”

As for the concrete molecular mechanism of the role of “gut microbiota-circadian clock axis” in metabolism, a growing amount of evidence demonstrated that microbiota-derived metabolites might play a crucial role. Leone et al. detected the intestinal microbial metabolic products, SCFAs, and H2S production in mice fed the low-fat diet or high-fat diet and showed that the metabolites also exhibited rhythmic fluctuations, particularly the butyrate. They found that the HFD led to significant alterations in microbial composition and circadian oscillations, as well as the bacterial metabolic products. Further adding the butyrate to a hepatic model in vitro significantly influenced the expression of *Bmal1* and *Per2*. Thus, bacterial metabolites might be a crucial mediator between gut microbiota and circadian clock [80]. Subsequently, Tahara et al. also confirmed the day-night differences of gut microbial-dependent SCFAs in mice, especially the acetate and butyrate. In addition, they also found that oral administration of SCFAs can result in dramatic changes of clock genes in peripheral clocks [83]. In addition to SCFAs, bile acid is found to be another factor participating in crosstalk between microbiota and circadian rhythms. Bile acids are synthesized from cholesterol moieties and are conjugated to taurine or glycine. Then, the conjugated bile acids were deconjugated by microbiota in the intestine. Govindarajan et al. showed that microbe-derived unconjugated bile acids can alter the expression of clock genes in the ileum, colon, and liver [84]. Therefore, adverse nutritional environment affects the structure and function of gut microbiota, and the changed microbial metabolites can further influence the circadian clock and metabolic health. The “gut microbiota-circadian clock axis” disorders may be a key mechanism by which adverse nutritional environments lead to abnormal glucose metabolism [80].

We previously reviewed the role of gut microbiota in the effects of maternal obesity on metabolism of offspring and indicated that gut microbiota might be an essential programing factor for the increased risk of metabolic disorders in later life induced by adverse nutritional environments in early life [85]. Our research [7] and other studies [86–89] have found that the composition and diversity of gut microbiota changed significantly, which was accompanied by metabolic disorders in later life, after exposure to adverse nutritional environments in early life. An animal experiment has shown that alpha diversity was significantly decreased in the gut microbiota of mice from HFD-fed dams compared with the chow diet-fed dams [9]. Another fecal transplantation study found that the beta diversity of intestinal microbiota in offspring from dams transplanted microbiota from mice-fed HFD was lower than that from the mice-fed normal diet, and the number of Firmicutes phylum was also decreased, which could produce butyrate [90]. Furthermore, Ma et al. demonstrated that the relative abundance of *Campylobacter* was persistently decreased until juveniles in primates from HFD fed mothers compared with that from the normal group [88]. Human studies also indicated that maternal HFG or obesity can significantly reduce the abundance of *Bacteroides*, *Blautia* spp., and *Eubacterium* spp. and increase the numbers of *Parabacteroides* spp. and *Oscillibacter* spp., which have been found to be associated with obesity previously [91–93]. In addition, evidence from human studies and animal models showed that early-life antibiotics use, which led to imbalance of gut microbiota, could result in long-term deleterious effects on health, including obesity and diabetes mellitus [94–96]. Thus, the changes of gut microbiota play important roles in linking adverse early-life exposures and metabolic disorders in later life.

In light of the important roles of circadian rhythm and gut microbiota, respectively, in early-life nutrition and metabolic health in later life and the close communication between gut microbiota and circadian clock, we propose that the “gut microbiota-circadian clock axis” might be a novel and crucial mechanism for deciphering “metabolic memory.” Adverse early-life exposures can significantly alter the composition and function of gut microbiota and associated microbe-derived metabolites, which further regulate the circadian clock and metabolism in peripheral tissues. However, the evidence is still scarce and more related studies need to be done in the future.

## 5. Conclusions and Prospects

The overall health resources for chronic diseases in China, and throughout the world, are mostly allocated to treating diseases or complications that have already developed. Thus, the prevention, early identification, and early treatment of diseases are extremely urgent. The momentum of DM development is impossible to stop in the short term, and it is necessary to adjust the prevention and control strategies in a timely manner. It is important to note that the prevalence of DM in adolescents has increased significantly. However, the inherent genetic factors and traditional environmental factors cannot completely explain the rapid increase in the prevalence of diabetes. What is the best way to identify groups that are at high risk of developing diabetes early? How can an earlier intervention be carried out? How can treatment effectiveness be improved? These are key issues in the prevention and treatment of metabolic diseases such as DM. Early detection of these problems with the philosophy that a “Good doctor treats when the disease does not appear” can generate twice the results with half the effort. Therefore, exploring the specific mechanism of “metabolic memory” in terms of the adverse nutrient environment in early life and identifying the key mechanism underlying the rapid increase in the development of DM may have far-reaching effects on reducing the barriers to the prevention and treatment of metabolic diseases.

**Figure 1:**
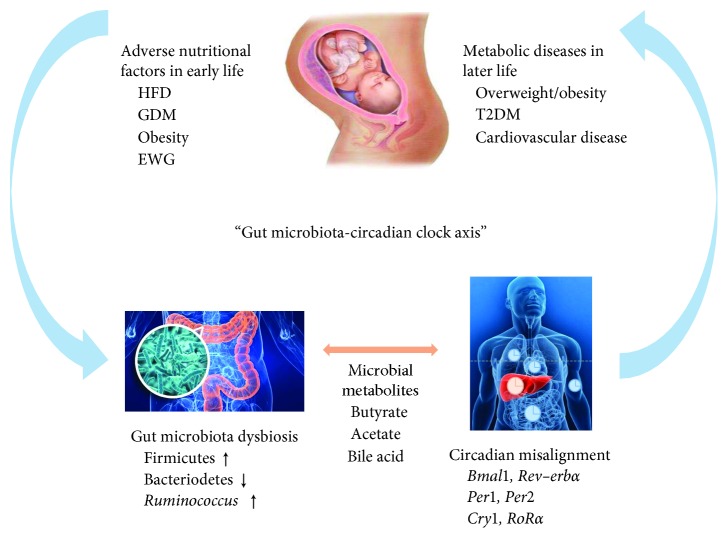
Overview of the role of “gut-microbiota-circadian clock axis” in the effects of adverse early-life nutritional environment on the metabolism in later life. HFD, high-fat diet; GDM, gestational diabetes mellitus; EWG, excess weight gain; T2DM, type 2 diabetes mellitus.

A multitude of studies have shown that a maternal HFD during pregnancy and lactation could increase the risk of developing obesity, impaired glucose tolerance, insulin resistance and fatty liver in offspring in early life, and these health problems can continue through adulthood and even to old age. Therefore, the adverse nutritional environment in early life can significantly increase the risk of abnormal glucose metabolism in later life. However, the concrete mechanism remains largely unclear. With the prevalence of shift working and jet lag, the role of circadian misalignment in metabolic diseases gained increasing attention. Emerging studies have shown that there were significant relationships between nutrient intake and circadian clock, especially in the early life. In addition, it is well known that adverse nutritional environment affects the structure and function of gut microbiota. Recently, several experimental animal models indicated that there was bidirectional communication between gut microbiota and circadian clock and the changed microbial metabolites could further influence the circadian clock and metabolic health. Thus, the intestinal microbial community plays a pivotal role in the adverse nutrient environment and circadian rhythm disorders. Based on the abovementioned fact, we propose that the disorder of the “gut microbiota-circadian clock axis” plays an important role in the “metabolic memory” (Figure 1).

However, studies clarifying the specific mechanism of “gut microbiota-circadian clock axis” and the cause and effect of the changes of metabolic health in later life and the axis are still limited, although they are of great significance for the prevention and early treatment of T2DM. Thus, there is an urgent need for more studies to further explore the underlying mechanisms. This review proposes a novel mechanism to decipher the “metabolic memory” and combine gut microbiota and circadian rhythm to reveal the effects of early-life adverse nutritional environments on glucose metabolism in adulthood. In this way, we can prevent diabetes in the high-risk population early and provide corresponding interventions to diabetic patients. This concept is expected to provide an important theoretical basis and a new drug target during the early stage of life to effectively prevent the occurrence and development of T2DM.

## Figures and Tables

**Table 1:** The summarization of the relationship between gut microbiota and circadian clock.

Conditions	Gut microbiota	Circadian clock	Reference
Jet lag	Loss of oscillations in *Ruminococcaceae*	*Bmal*, *Rev-erbα*, and *RORγt*	[66]
High-fat, high-sugar diet	Increase in ratio of Firmicutes/Bacteriodetes and *Ruminococcus*	*Per2*	[69]
Cyanobacteria	*Synechococcus elongatus PCC 7942*	kaiA, kaiB, and kaiC	[70]
Human	*Enterobacter aerogenes*	Melatonin	[72]
Antibiotic treatment or germ-free mice	Antibiotic-induced gut microbial alterations or the absence of gut microbes	*RORα*, *RevErbα*, *Bmal1*, *Cry1*, *Per1*, and *Per2*	[73, 74]
Fecal transplantation from high-fat-diet-fed donors	Increase in Firmicutes and decrease in Bacteriodetes	*Bmal1*, *Per2*, *Rev-erba*, and *Dbp*	[75]
